# Swarm intelligence-based model for improving prediction performance of low-expectation teams in educational software engineering projects

**DOI:** 10.7717/peerj-cs.857

**Published:** 2022-01-19

**Authors:** Bilal I. Al-Ahmad, Ala’ A. Al-Zoubi, Md Faisal Kabir, Marwan Al-Tawil, Ibrahim Aljarah

**Affiliations:** 1 Faculty of Information Technology and Systems, University of Jordan, Aqaba, Aqaba, Jordan; 2 School of Science, Technology and Engineering, University of Granada, Granada, Spain, Spain; 3 King Abdullah II School for Information Technology, University of Jordan, Amman, Ãmmãn, Jordan; 4 Pennsylvania State University - Harrisburg, Middletown, PA, USA; 5 United International University (UIU), Dhaka, Bangladesh

**Keywords:** Optimization, PSO, Software engineering, Data mining, Machine learning, Artificial intelligence

## Abstract

Software engineering is one of the most significant areas, which extensively used in educational and industrial fields. Software engineering education plays an essential role in keeping students up to date with software technologies, products, and processes that are commonly applied in the software industry. The software development project is one of the most important parts of the software engineering course, because it covers the practical side of the course. This type of project helps strengthening students’ skills to collaborate in a team spirit to work on software projects. Software project involves the composition of software product and process parts. Software product part represents software deliverables at each phase of Software Development Life Cycle (SDLC) while software process part captures team activities and behaviors during SDLC. The low-expectation teams face challenges during different stages of software project. Consequently, predicting performance of such teams is one of the most important tasks for learning process in software engineering education. The early prediction of performance for low-expectation teams would help instructors to address difficulties and challenges related to such teams at earliest possible phases of software project to avoid project failure. Several studies attempted to early predict the performance for low-expectation teams at different phases of SDLC. This study introduces swarm intelligence -based model which essentially aims to improve the prediction performance for low-expectation teams at earliest possible phases of SDLC by implementing Particle Swarm Optimization-K Nearest Neighbours (PSO-KNN), and it attempts to reduce the number of selected software product and process features to reach higher accuracy with identifying less than 40 relevant features. Experiments were conducted on the Software Engineering Team Assessment and Prediction (SETAP) project dataset. The proposed model was compared with the related studies and the state-of-the-art Machine Learning (ML) classifiers: Sequential Minimal Optimization (SMO), Simple Linear Regression (SLR), Naïve Bayes (NB), Multilayer Perceptron (MLP), standard KNN, and J48. The proposed model provides superior results compared to the traditional ML classifiers and state-of-the-art studies in the investigated phases of software product and process development.

## Introduction

Data mining and machine learning have been used in several studies (Naidu et al., 2020; Pérez & Rubio, 2020) to achieve successful education development. Educational data mining (Bacos, 2019) is key technique that aims to get better educational practices and improves learning outcomes. Software engineering (Akhlaq & Yousaf, 2010; Hayes et al., 2016; Rubinstein, 2007) aims to understand the overall SDLC to increase the poor-quality of software product. Educational software engineering (Ömeroğulları, Guill & Köller, 2020; Bavota et al., 2012) is an academic field that extensively explores several aspects about software product and process. The software project is an essential part of the software engineering course and it consists from both software product and process parts. The software product is the final product that resulted from adapting the software process. The software process involves following specific practices and behavior while working in software project. Software projects have characteristics for each of software product and process. Usually, software projects are evaluated at theoretical and technical perspectives. The theoretical part reflects the software process aspects while the technical part is relevant to the software product aspects.

Asuncion & Newman (2007) concluded that measuring such characteristics during the software engineering course is very important to assess the performance of software teams and eventually achieve better learning outcomes. The software product features capture software product deliverable as user interface, performance, architecture, database design, code, and presentation for final delivery. Besides, the software process features capture certain team activities and practices like quality, meetings participation, and time completeness for the software deliverables.

Project-based learning is an important teaching method in software engineering education. Software projects learning (Adriano, 2019; Dingsøyr et al., 2018) is a key task in software engineering and it is hard to evaluate software projects. Software projects have teams, teams shall have certain skills to learn the proper activities and practices related to software project development. Students who are working in teams face some challenges related to their performance, cooperation, delivery deadline, *etc*. So, detecting these problems at early phases of software project would effectively help low-performing teams to overcome these challenges.

There are a number of studies (Menezes, Gusmão & Moura, 2019; Bajwa et al., 2017) discovered project failure analysis and prediction from an industrial aspect. Other studies (Gulati & Sharma, 2020; Sauer & Cuthbertson, 2003; Sauer, Gemino & Reich, 2007; Daughtrey, 2014; Reel, 1999; Charette, 2005; Duhigg, 2016; Menezes, Gusmão & Moura, 2019) explored the software engineering project failure in the educational perspective. Gulati & Sharma (2020) pointed that the prediction performance of teams in the project-based environment is a demanding task due to the existence of various difficulties like team distribution, time zone, and physical distance. Moreover, some studies (Sauer & Cuthbertson, 2003; Sauer, Gemino & Reich, 2007; Daughtrey, 2014; Reel, 1999; Charette, 2005; Duhigg, 2016) presented the causes of software project failures such as: teamwork failures, lack of experience, schedule delay, and global distribution perspective. In addition, Menezes, Gusmão & Moura (2019) stated other factors that might contribute to software project failure such as imprecise project goals, poor teamwork communication, and incorrect project requirements.

The prediction of performance for students teams in software project is time-consuming to conduct manually. So, there are several studies (Fitzgerald, Letier & Finkelstein, 2011; Manalif et al., 2012; García et al., 2017; Hale, Jorgenson & Gamble, 2011; Raza, Faria & Salazar, 2019; Nguyen & Chua, 2016) which proposed various tools for tracking the performance of students in software projects. Fitzgerald, Letier & Finkelstein (2011) presented a tool that automated failure analysis models by employing ML classification algorithms to evaluate students performance. Manalif et al. (2012) presented different evaluation tools for project risk that used the flippant fuzzy reasoning with the aid of the professional COCOMO. Further, García et al. (2017) used information technology tools for evaluating project performance through integrating with ML approaches to better deal with imprecision issues that affect the project-learning evaluation. Moreover, an integrated course-ware tool called (SEREBRO) is presented in Hale, Jorgenson & Gamble (2011) which allows the instructor to visualize and provide feedback in the ongoing software projects. Similarly, Raza, Faria & Salazar (2019) presented a tool called (ProcessPAIR) which used to automate the performance analysis of students in software development projects. Also, another prediction tool is presented by Nguyen & Chua (2016) that used to provide a performance feedback on how each team’s member is contributing in software project. Le, Chua & Wang (2017) supported a solution tool that utilizes Git-driven technology to measure team contributions in software engineering projects.

Predicting the performance of students teams (Fitzgerald & Stol, 2017) has increased attention to improve learning techniques in software engineering education. The early prediction of low-performing teams reduces the possibilities of project failure which is considered a major challenge against successful software engineering. Several studies (Petkovic et al., 2014; Petkovic et al., 2016; Petkovic et al., 2018; Al-Taharwa, 2020; Naseer, Zhang & Zhu, 2020a; Naseer, Zhang & Zhu, 2020b) attempted to early predict the performance of students teams in software engineering education by implementing different classification models and features selection techniques by demonstrating different phases of software project.

Feature selection is an urgent task that should be applied to handle difficulties related to classification models. The high dimensional data increased speedily and enforced significant challenges on the existing classification methods as it caused slow learning of model. Therefore, it becomes a necessity to know how to handle this type of data. The optimal way to deal with such data is to perform a feature selection technique. Feature selection can be defined as a pre-step task to select the best subset of features and to remove the redundant and irrelevant features. This study utilizes a metaheuristic algorithm as a features selection technique to decrease cost and time while running classifiers, and increase the chances to detect the most relevant features which lead to increase the performance of ML classifiers. Such improvement happened based on utilization of the wrapper features selection method that follows the evaluation criteria at each selection. The proposed model uses one of the most effective feature selection technique (PSO) which helps to detect the most relevant product and process features.

This study uses PSO as feature selection method, and the KNN as classification model to introduce an swarm intelligence -based model (PSO-KNN) to improve the predicting performance of low-expectation teams at the earliest possible phases of software product and process development. The proposed model investigates the first five time intervals (T1 to T5) as training set, and utilizes the last time interval (T11) as a testing set. During each phase, the results are tracked to detect the earliest possible phase of SDLC to early predict the final grade for these certain types of teams. Capturing the most relevant features significantly improves the prediction accuracy for the (KNN) classifier. As a result, using such combination between PSO and KNN would improve the prediction performance for the low-expectation teams instead of using traditional ML classifiers. Experiments were conducted on SETAP project dataset and compared with different traditional ML classifiers and related studies. The proposed model outperforms others ML classifiers in the investigated phases for software product and process development.

The motivation of this study is to improve the prediction performance for low-expectation teams at the earliest possible phases of SDLC. The proposed model helps to improve the learning practices and outcomes of software projects by encouraging the low-expectation teams to progress in a more efficient manner and prepare them to better move for the software industry in their future careers. Using the proposed model would be valuable to better understand the intersection points between software engineering education and software industry. Furthermore, the proposed model has significant implication at theoretical (*i.e.,* software process) and practical (*i.e.,* software product) aspects of the software project. Also, the proposed model helps to decrease the rate for the project failure by bridging the gaps and miscommunication issues between instructors and students, and helps instructors to have better assessment strategies for the low-expectation teams through tracking their future progress during different phases of software project.

Further, the proposed model helps to achieve cooperative learning among teams to make better academic achievements. By using the proposed model, software engineering instructors can track the performance of low-expectation teams at the product development through checking the software deliverable that related to requirements, design, coding, testing, and *etc*. With respect to software process, software engineering instructors can detect the dynamic behavior and practices of such teams while they work on each phase of SDLC. Capturing both perspectives of software product and process would allow instructors to predict the performance of low-performing teams at the earliest possible phases of SDLC by early detecting the concerns and challenges that affect the overall project progress such as late submissions, miscommunication, and incorrect software deliverables.

The limitations of this study could be declared as follows: (1) It is difficult to generalize the reported findings by the proposed model on such a one type of datasets, other types of dataset was difficult to obtain in software engineering education; (2) another limitation was that the proposed model demonstrated the experiments of software product and process parts separately, in the case of combining the product and process features together, the prediction performance of the proposed model might be affected.

The remaining parts of the paper is structured as follows: A brief summary of the relevant studies is given in Related Work. Materials and Methods presents the dataset and the proposed methodology used in this research. Experiments and Results describe the obtained results at both software product and process development. The Discussion for the outcomes is described as in discussion section. The limitations of this study is declared in the Limitations section. Also, the findings of this research and future works directions are stated in the Conclusion and Future Work section.

## Related Work

Several approaches (Kotsiantis, 2012; Lykourentzou et al., 2009; Castro et al., 2007; Baker & Yacef, 2009; Baker, 2000; Macfadyen & Dawson, 2010; Delen, 2010; Jovanovic et al., 2012; Hu, Lo & Shih, 2014; Guo et al., 2015) used various ML approaches to assess individual performance of students by investigating personal and quantitative academic aspects such as grades, dropping frequency, teaching effectiveness, and e-learning techniques. Similarly, some other studies (Zafra & Ventura, 2009; Thai-Nghe, Horváth & Schmidt-Thieme, 2010) used traditional methodologies such as genetic programming and task recommendation systems to evaluate the performance of students in software projects. Nevertheless, these studies used specific attributes such as quizzes, assignments, attendance, and GPA to evaluate the students’ performance. Another study (Abidin et al., 2019a) was conducted to explore individual performance of students in software engineering courses using MLP approach. Moreover, Le, Chua & Wang (2017) used text similarity and machine learning to construct formative evaluation for students in software projects. These studies explored the performance based on individual assessment instead of considering the teamwork assessment. In contrast, the proposed model explores the teamwork assessment which is very essential key to predict the overall performance in project-learning environment of software engineering.

The research studies (Petkovic et al., 2014; Petkovic et al., 2016; Petkovic et al., 2018; Al-Taharwa, 2020; Naseer, Zhang & Zhu, 2020a; Naseer, Zhang & Zhu, 2020b) aimed to early predict performance of low-expectation teams by utilizing the SETAP project dataset, they applied various classification and features selection techniques on different time intervals of software project.

Some studies (Petkovic et al., 2014; Petkovic et al., 2016; Petkovic et al., 2018) used Random Forest (RF) classifier and the GINI index as feature selection technique to early predict performance of students teams. These studies considered second time interval (T2) for software process and the third time interval (T3) for software product to report their results. These studies investigated single phases of software product and process development instead of capturing the continues phases of software product and process development. They used these particular phases in a purpose of guiding software engineering instructors to implement an early intervention strategy at the early design and implementation phases to avoid the project failure for low-expectation teams.

Al-Taharwa (2020) used decision tree classifier on the second and sixth time intervals (T2 and T6) for software process, and the third and seventh time intervals (T3 and T7) for software product to early predict the software project failure by focusing on teamwork distribution. His approach used those time intervals to increase the ability to predict the dynamical behaviors of software teams at design and implementation phases of SDLC.

In addition, Naseer, Zhang & Zhu (2020b) used software product dataset to predict coding intricacy through highlighting code deliverable characteristics by employing LogitBoost, AdaBoost, Bagging, Random forest, J48, sequential minimal optimization (SMO), multilayer perceptron (MLP), and Naïve Bayes (NB) classifiers, they used the last time interval (T11) as training and testing set as it covered composite aspects of implementation through final delivery phases.

Further, Naseer, Zhang & Zhu (2020a) employed J48 as a classifier and information gain as feature selection technique on software product dataset to early predict the performance of low-expectation teams at the essential phases of SDLC. The study utilized the first five time intervals (T1, T2, T3, T4, T5) as training set and the last time interval (T11) as testing set. However, the study captured only software product development rather than process development. This study captured both of software product and process development. Exploring software process development is an important method to track the behavior of low-expectation teams at the main phases of SDLC. As Naseer, Zhang & Zhu (2020a) utilized the same training and testing set like this study, we compared the results achieved by the proposed model with their findings in particular to software product development.

This study introduces an swarm intelligence based model (PSO-KNN) which aims to improve the performance prediction of low-performing teams at the earliest possible phases of SDLC, and it attempts to reduce the selected software product and process features. This paper uses PSO as feature selection method and the KNN as classification model, and it captures both software product and process perspectives to investigate the continues phases of SDLC (*i.e.,* requirements analysis, design, implementation, testing, and maintenance). During each phase, the obtaining results are tracked to detect the earliest possible phase of software development to predict the final grade for low-expectation teams.

## Materials and Methods

The section contains the following subsections: description of the used dataset, features selection, PSO, background about (PSO-KNN), the proposed methodology, and evaluation measures which have been applied in this research study.

### Dataset description

The dataset was developed in the SETAP project during the fall semester of 2012 to 2015 for 74 different students-teams in software engineering course. The SETAP project collects the objective and quantitative data through a joint project among San Francisco State University (SFSU), Fulda University (Fulda), and Florida Atlantic University (FAU). The dataset contains 115 software product features and 84 process features, and these features are collected using various data sources like weekly time cards, tool logs, class data, and instructor observations as declared in Petkovic et al. (2014). Weekly time cards are used to collect information from each single student about the time spent on coding, meeting, and teamwork activities during each week. Tool logs is used to collect statistics information about usage of cooperation and development tools like total number of e-mails between the members of each team, frequencies of postings to source code repository, and number of commit messages. Class data is used to collect general information such as year, semester, team leader gender, team-ID, and team distribution (*i.e.,* local or global). Instructor observation is used to help instructors record information about different activities as team participation, number of instructor intervention, and number of timely delivered issues.

Software product features represent software deliverables at each phase of SDLC while software process features capture the activities and dynamic behaviors of software teams during SDLC. There are 11 time intervals (T1 through T11) of assessments, and each team is assessed at these different time intervals to predict the performance of teams during software project. Moreover, each student’s team is graded as A (high expectation) or F (low expectation) for each time interval. The first five-time intervals (T1, T2, T3, T4, and T5) represent the key phases of SDLC. The remaining time intervals(T6 through T11) are aggregated-tasks and they are combined from different sets of the first five-time intervals (T1 through T5). The goal of such aggregation is to track the dynamical behavior of students teams during the software development life cycle. For example, the eleventh time interval (T11) represents the last phase, and it combines information from the third time interval until the fifth time interval (T3 through T5). The final phase records the behavior of teams during coding, testing, and final product delivery phases. Further description of the dataset and features are provided by Petkovic et al. (2016).

### Features selection

The classification models depend on high-quality training data. If the data suffer from redundant and useless information, then it can lead to adverse outcomes from time to time (Al-Qudah et al., 2020a; Ala’M et al., 2021; Srinivasan et al., 2021; Al-Zoubi et al., 2021; Faris et al., 2017; Al Qudah et al., 2020b). Recently, the high dimensional data increased promptly and enforced significant challenges on the existing classifier methods (Obiedat et al., 2021). For instance, it may be caused performance to degenerate, interpret difficulty, and slow learning of model. Therefore, it becomes a necessity to know how to handle this type of data. The optimal way to deal with such data is to perform a feature selection technique. Feature selection (Kabir & Ludwig, 2019a; Al-Ahmad, 2018) can be defined as a pre-processing operation in order to select the best subset of features and to remove the redundant, noisy, and irrelevant ones.

Mainly, there are two mechanisms of feature section techniques, filter and wrapper feature selection. The filter mechanism is more about the relation or correlation between the features without considering the class label, whereas the wrapper feature selection depends entirely on the class label to select the most important features. The wrapper technique can be employed using metaheuristic algorithms, such as Genetic Algorithm (GA) (Al-Qudah et al., 2020a; Al-Qudah et al., 2020b), Particle swarm optimization(PSO) (Rostami et al., 2020), Multi-Verse Optimizer (MVO) (Sadiq et al., 2019), Salp Swarm Algorithm (SSA) (Ala’M et al., 2020), Whale Optimization Algorithm (WOA) (Ala’M et al., 2018) and Competitive Swarm Optimizer (CSO) (Ala’M et al., 2021). To accomplish the feature selection task, Petkovic et al., (2014) and Petkovic et al. (2016) used GINI index whereas Naseer, Zhang & Zhu (2020a) used information gain. In contrast, the proposed model uses more effective feature selection (PSO), which helps to reduce the number of the selected product and process features that improve the performance of early prediction for low-expectation teams.

### Particle swarm optimization

Particle Swarm Optimization is one of the most popular metaheuristic algorithms that stimulated the natural behavior of flock birds (Kennedy & Eberhart, 1995). In other words, PSO inspired the position of these birds to obtain the optimal solution. Particles, in PSO known as the population, are a set of solutions denoted by the various individuals in multidimensional space. The particles (population) gathered together to form a swarm shape in charge of searching the space at a particular velocity for the optimal solution.

Furthermore, the population of PSO has the capability to remember and recall the previous best solution (position). Such positions of the population can be modified and determined the optimal solution from all suggested solutions based on the pbest, personal best experience, and gbest, global best, of the PSO (Marini & Walczak, 2015). The historical behavior of the population and their near particles assist in updating the velocity during the searching phase. Hence, we can notice the improvement of the searching process in every iteration as stated in Ghamisi & Benediktsson (2014). The PSO updates its position based on the following equations:

(1) \begin{eqnarray*}{X}_{i}(t+1)={X}_{i}(t)+{V}_{i}(t+1)\end{eqnarray*}

where *X*_*i*_ denotes the particle position *i*, while *t* is the iteration number. The velocity indicates with *V*_*i*_ of particle *i*.

(2) \begin{eqnarray*}{V}_{i}(t+1)=W\cdot {V}_{i}(t)+{r}_{1}\cdot {c}_{1}\cdot [pBes{t}_{i}-{X}_{i}(t)]+{r}_{2}\cdot {c}_{2}\cdot [gBes{t}_{i}-{X}_{i}(t)]\end{eqnarray*}

where *W*, *r*_1_, and *r*_2_ is the inertia weight and the numbers between 0 and 1, respectively. The constant coefficients are *c*_1_ and *c*_2_, while *pBest*_*i*_ denotes the current best position at particle *i* and the current global best position is *gBest*_*i*_ of the particle’s neighbors.

In this study, the PSO is combined with one of the well-known classification model, namely, K-Nearest Neighbors (KNN) to improve the prediction performance for low-expectation teams at the earliest possible phases of SDLC. Moreover, the study attempts to select the most relevant software product and process features that improve the prediction performance for such teams.

### Background about PSO-KNN

The KNN classifier is one of the most common used classifiers as it is simple and effective non-parametric approach for classification (Gweon, Schonlau & Steiner, 2019). It has one parameter (K) to identify the number of selected nearest neighbors (Quiros et al., 2017) to predict the class labels of the unknown samples. The value of this parameter has a significant impact on classification performance (Zhang et al., 2018). However, searching for the value of (K) is difficult, especially with high-dimensional data. Generally, the K parameter in the KNN classifier is selected empirically. Depending on each problem domain, various numbers of nearest neighbors are tested, and the parameter with the best accuracy is chosen to define the classifier. Several studies (Tharwat et al., 2018; Bui et al., 2019; Ni et al., 2012; Abidin et al., 2019b; Zyout, Abdel-Qader & Jacobs, 2011) utilized the strength of combination between PSO and KNN to improve the prediction accuracy in different problems domains. Tharwat et al., (2018) proposed the PSO-KNN model to predict human activities in a mobile crowd-sensing environment. Bui et al. (2019) proposed a hybrid technique (PSO-KNN) for estimating blast-induced ground vibration. Another study (Abidin et al., 2019b) proposed a PSO-KNN model for object position estimation system in the room. Also, Ni et al. (2012) applied (PSO-KNN) approach to predict the cycle time of wafer fab lots. Finally, Zyout, Abdel-Qader & Jacobs (2011) presented a (PSO-KNN) approach to classify the micro-calcification clusters in mammography.

Likewise, this study utilizes the strength of combination between PSO and KNN. The individual encoding of each solution uses PSO to search for the most relevant features by employing binary evaluation. Before implementing any meta-heuristic algorithms, it is an important to take into account two main issues, individual encoding and determining the fitness function. In this study, the individual encoding consists of binary values 0 and 1, describes the selection of the best subset of features. In other words, the solution (particles) that is provided randomly by the PSO is represented by a one-dimensional vector that portrayed the attributes (features) of the original dataset. The values of the vector are rounded; if the value is equal to or over 0.5, it means that the feature was selected and rounded to 1, otherwise the feature will not be selected and that vector rounded to 0. As for the second issue, the fitness function is applied to evaluate the particles (solutions) provided by the PSO algorithm. The assessment of each particle quality is performed by the KNN. This paper used the accuracy measure as a fitness function that can be calculated according to Eq. (3). Moreover, the PSO attempts to increase the accuracy value to obtain the best possible results.

**Figure 1:**
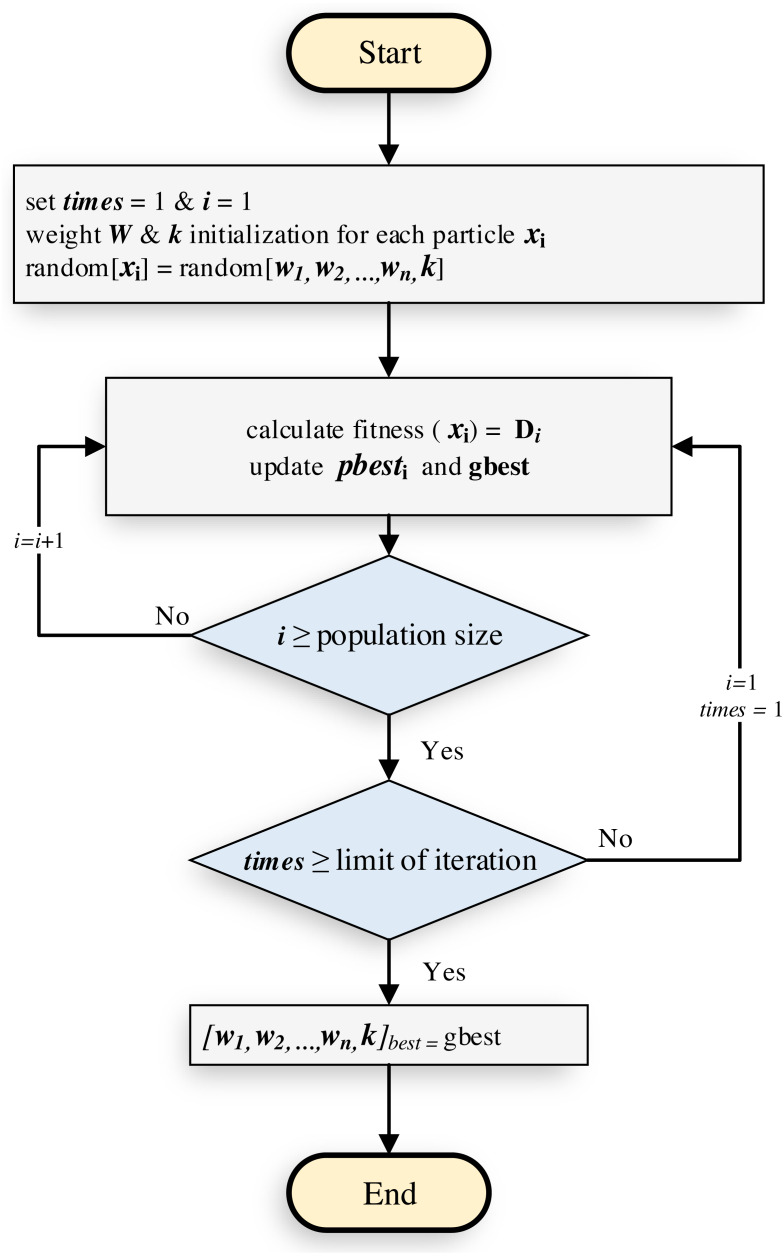
PSO initialization process.

**Figure 2:**
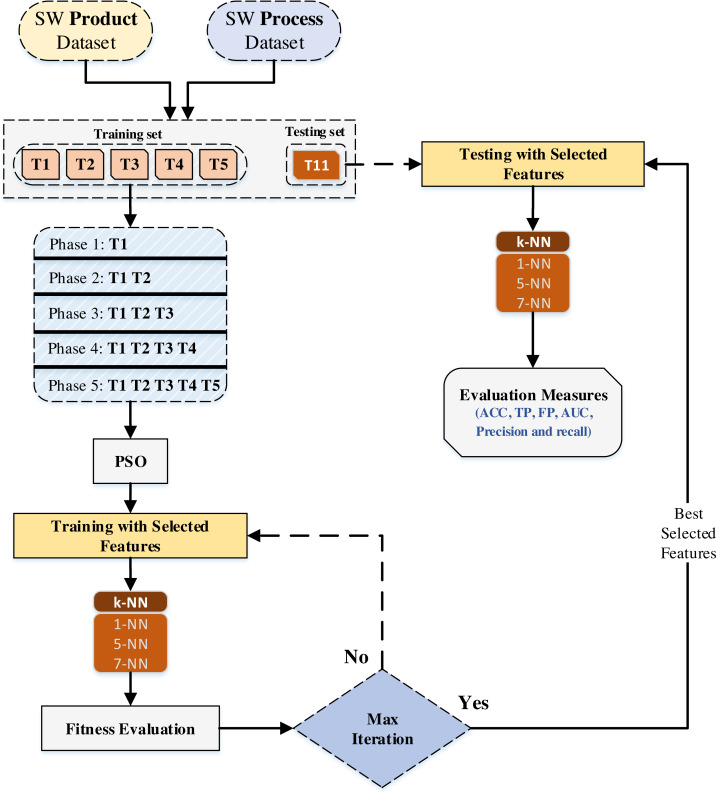
Overview of the proposed assessment model.

(3) \begin{eqnarray*}fitness({I}_{i}^{t})= \frac{1}{K} \sum _{k=1}^{K} \frac{1}{N} \sum _{j=1}^{N}\delta (c({x}_{j}),{y}_{j})\end{eqnarray*}

where the accuracy outcome indicates by *c*(*x*_*j*_) and *y*_*j*_ is the actual label of the *j*th instance, while *δ* is the correlation between *c*(*x*_*j*_) and *y*_*j*_. Thus, when *c*(*x*_*j*_) equal *y*_*j*_, then *δ* = 1, if not *δ* = 0. The number of instance denotes by *N* and *K* is the number of folds. shows the PSO initialization process. The PSO is initialized with a population of random solutions and searches for optima by updating generations as illustrated in Fig. 1. Moreover, PSO achieves maximum performance when it is implemented in the low dimensional search space as the data in SETAP project. The performance of PSO (Yang et al., 2013) extensively depends on the initialization of the swarms. The overall methodology is depicted as in Fig. 2.

### The proposed methodology

The swarm intelligence-based model is proposed to improve the prediction performance for low expectation-teams at the earliest possible phases of SDLC. The PSO is applied for feature selection, and the KNN is used for classification. The features selection is implemented to identify the most relevant features of software product and process that improve the prediction performance for the low-expectation teams. The classification technique is used to predict the performance for low-expectation teams at the earliest possible phases of SDLC.

The proposed model begins by splitting the datasets (software product and software process) into training and testing subsets. The training set comprises several different datasets from the two types, including T1, T2, T3, T4, and T5, while the testing set consists of T11 only. In the training task, we generate the five phases (phase 1, phase 2, phase 3, phase 4, and phase 5) from the trained data, namely, (T1), (T1 & T2), (T1, T2 & T3), (T1, T2, T3 & T4), and (T1, T2, T3, T4 & T5). Each of which is tested against the phase 11 to examine the performance prediction of every phase of SDLC. Then, during the training stage, PSO will be implemented for feature selection.

This paper uses the PSO-KNN framework to reduce the feature space dimensions, select the optimal subset of features, and tune the classifier parameters in order to improve the generalization ability of the classification process. Moreover, PSO-KNN optimizes the performance of the KNN classifier by finding the best value of (K) that produces the best classification performance. Among the population-based optimization algorithms, PSO is extensively used to optimize parameters in pattern recognition tools as they do not suffer from local minima problems as declared in Sasirekha & Thangavel (2019).

This research work justifies the implementing of combination between PSO and KNN based on various reasons. First, the PSO is a well-regarded choice of algorithms for solving hard-optimization problems in a reasonable amount of time. Second, the KNN is simple to use and easy to understand as a nonparametric learning algorithm and it has very flexible decision boundaries. Third, this paper followed the suggestions of many research works as Wang et al. (2015) which utilized the KNN as a classifier because it is considered an instance-based or memory-based learning techniques. Fourth, the whole computation of KNN is executed during classification, and there is no need for training task to construct the classifier. Thus, KNN is a very efficient and simple algorithm, it takes a O(1) as a time complexity for training step. Moreover, the time complexity of O(mn + mlog2m) is given to classify a new instance in the training set with (m) instances and (n) attributes, where O(mn) represents the time needed to compute the distances between new instance and each single instance in the training set, and O(mlog2m) indicates the time required to sort all the distances between new instance and it’s k-nearest neighbors.

Selecting the optimal (K) is a common difficult task for variety of problems as pointed in Song et al. (2007), Zhang et al. (2017b) and Zhang et al. (2017a). As the performance of KNN classifier significantly differs when (K) is changed (Hassanat et al., 2014). Nevertheless, it is shown in the literature (Latourrette, 2000) that when the data is distributed in a uniformal way, determining the value of (K) in advance becomes difficult.

In the light of this study, three different values of K (*K* = 1, 5, and 7) were selected based on studying the literature and what most previous works used for the KNN classifier as recommended in the literature (Baldini & Geneiatakis, 2019; Zhang et al., 2017b; Huang et al., 2018; Zhang et al., 2018). Such selection is used to construct the three combinations like (PSO-1NN, PSO-5NN, and PSO-7NN) which reflect the best optimal selection for the performance predication based on our dataset distribution and experimental settings.

### Evaluation measures

To measure the performance of the proposed model, several evaluation metrics were used such as precision (Miao & Zhu, 2021), recall (Tharwat, 2020), F-measure (Soleymani, Granger & Fumera, 2020), accuracy (Dinga et al., 2019), and Area Under Curve (AUC) (Wang, Wong & Lu, 2020; Kabir & Ludwig, 2019b). These were derived from the confusion matrix (Xu, Zhang & Miao, 2020; Markoulidakis et al., 2021) to calculate different evaluations for the proposed model. Also, This study uses the TP Rate and FP Rate as evaluation measures. The TP Rate is the ratio between the number of correctly classified students teams who failed in the project (low-expectation teams) and the total number of students that failed in the project per each team. The FP Rate is the ratio between the number of false positives and the total number of false positives plus the false negatives.

## Experiments and Results

This section describes the conducted experiments and the obtained results of this study, and it has two parts for each software product and softare process. The first part of our results presents the performance of the proposed model (PSO-KNN) comparing with other traditional ML classifiers, namely SMO, SLR, NB, MLP, and standard KNN. The second part describes the key software features obtained from each investigated phase as in the following subsections.

As PSO is randomized algorithm, it is an important to ensure the robustness of such algorithm by executing several 30 runs. The PSO-1NN, PSO-5NN, and PSO-7NN were constructed to report the best achieved results after comparing their performance values in both software product and process experiments. In terms of PSO, we employed the default values of PSO as suggested in the studies (He, Ma & Zhang, 2016; Boro & Bhattacharyya, 2017), the parameters setting of the PSO can be found in Table 1.

**Table 1:** PSO’s parameter settings.

Algorithm	Parameter	Value
PSO	Acceleration constants	[2.1, 2.1]
	Inertia w	[0.9, 0.6]
	Swarm size	30
	Number of itearions	100

### Software product results

This study aims to improve the prediction performance for low-expectation teams at the earliest possible phases of SDLC. The software product captures various types of software deliverable which should be submitted at each phase of SDLC. For the experiments on software product, this study demonstrate the experiments on the first five phases as training set incrementally, and the eleventh phase as testing set. This subsection compares the performance of the proposed model with machine learning techniques and the best results available so far in the literature.

The prediction outcomes of the investigated phases, starting from the first phase until the fifth phase are described below. Tables 2–6 shows the best performance achieved by the proposed model and other traditional ML models in the investigated phases.

**Table 2:** Performance of the classifiers in the first phase for software product.

Algorithm	TP rate	FP rate	Precision	Recall	F-Measure	AUC	Accuracy
SMO	0.125	0.119	0.444	0.125	0.195	0.503	55.41
SLR	0.125	0.119	0.444	0.125	0.195	0.597	55.41
NB	0.063	0.095	0.333	0.063	0.105	0.442	54.05
MLP	0.375	0.238	0.545	0.375	0.444	0.641	59.46
Standard KNN	0.500	0.405	0.485	0.500	0.492	0.548	55.40
PSO-1NN	**0.531**	0.238	**0.63**	**0.531**	**0.576**	**0.647**	**66.22**
PSO-5NN	**0.531**	**0.429**	0.486	**0.531**	0.507	0.557	55.41
PSO-7NN	0.375	0.405	0.414	0.375	0.393	0.519	50

**Notes.**

Numbers in bold indicate the best values.

**Table 3:** Performance of the classifiers in the second phase for software product.

Algorithm	TP rate	FP rate	Precision	Recall	F-Measure	AUC	Accuracy
SMO	0.438	0.167	0.667	0.438	0.528	0.635	**66.2**
SLR	**0.5**	0.214	0.64	**0.5**	**0.561**	**0.755**	**66.2**
NB	0.125	0.024	**0.80**	0.125	0.216	0.647	60.81
MLP	0.375	0.19	0.60	0.375	0.462	0.687	62.16
Standard KNN	0.438	0.262	0.560	0.438	0.491	0.588	60.81
PSO-1NN	0.375	0.143	0.667	0.375	0.48	0.6167	64.86
PSO-5NN	0.406	0.31	0.5	0.406	0.448	0.515	56.76
PSO-7NN	**0.5**	**0.333**	0.533	**0.5**	0.516	0.552	59.60

**Notes.**

Numbers in bold indicate the best values.

**Table 4:** Performance of the classifiers in the third phase for software product.

Algorithm	TP rate	FP rate	Precision	Recall	F-Measure	AUC	Accuracy
SMO	0.719	0.095	0.52	0.719	0.78	0.734	82.43
SLR	0.781	0.119	0.833	0.781	0.806	0.888	83.08
NB	0.5	0.095	0.8	0.5	0.615	0.813	72.97
MLP	0.625	0.048	**0.909**	0.625	0.741	0.903	81.8
Standard KNN	0.875	0.119	0.848	0.875	0.862	0.878	87.83
PSO-1NN	**0.906**	0.071	0.906	**0.906**	**0.906**	**0.917**	**91.89**
PSO-5NN	0.656	**0.143**	0.778	0.656	0.712	0.841	77.03
PSO-7NN	0.625	**0.143**	0.769	0.625	0.69	0.826	75.68

**Notes.**

Numbers in bold indicate the best values.

**Table 5:** Performance of the classifiers in the fourth phase for software product.

Algorithm	TP rate	FP rate	Precision	Recall	F-Measure	AUC	Accuracy
SMO	0.438	0.095	0.778	0.438	0.56	0.792	70.27
SLR	0.813	**0.71**	0.897	0.813	0.852	0.918	87.84
NB	0.438	0.095	0.778	0.438	0.56	0.792	70.27
MLP	0.906	0.214	0.763	0.906	0.829	0.926	83.78
Standard KNN	0.938	0.143	0.833	0.938	0.882	0.897	89.18
PSO-1NN	**0.969**	0.071	**0.912**	**0.969**	**0.939**	0.949	**94.59**
PSO-5NN	0.875	0.143	0.824	0.875	0.848	**0.952**	86.49
PSO-7NN	0.781	0.167	0.781	0.781	0.781	0.911	81.08

**Notes.**

Numbers in bold indicate the best values.

**Table 6:** Performance of the classifiers in the fifth phase for software product.

Algorithm	TP rate	FP rate	Precision	Recall	F-Measure	AUC	Accuracy
SMO	0.813	0.048	0.929	0.813	0.867	0.882	89.19
SLR	0.813	0.167	0.788	0.813	0.8	0.935	82.43
NB	0.813	0.214	0.743	0.813	0.776	0.905	79.73
MLP	0.813	0.048	0.929	0.813	0.867	0.95	89.19
Standard KNN	0.938	0.119	0.857	0.938	0.896	0.909	90.54
PSO-1NN	**0.969**	0.119	0.861	**0.969**	**0.912**	0.925	**91.89**
PSO-5NN	0.781	0.071	0.893	0.781	0.833	**0.96**	86.48
PSO-7NN	0.781	**0.24**	**0.962**	0.781	0.862	0.919	89.19

**Notes.**

Numbers in bold indicate the best values.

**Figure 3:**
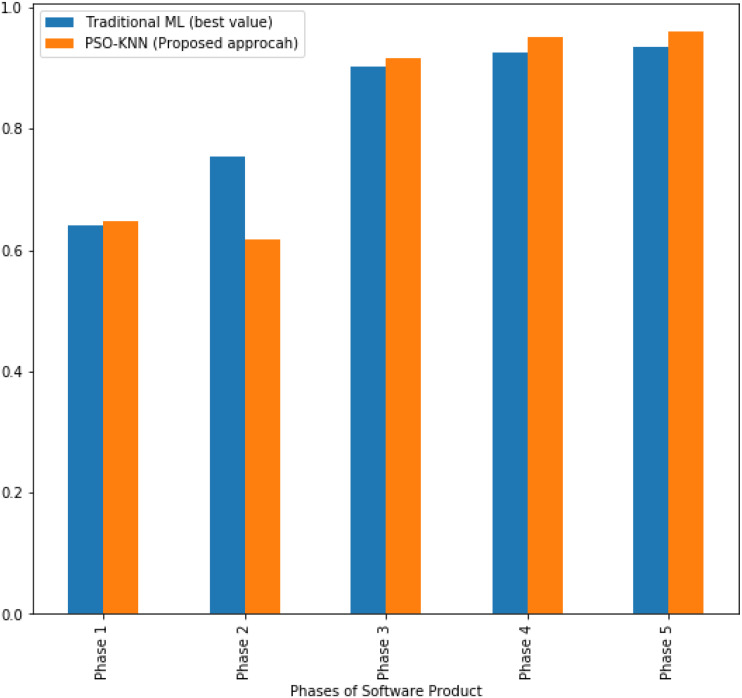
The AUC values obtained by the proposed model and the best values achieved by ML techniques for software product.

Considering the investigated phases, the best recall values achieved by ML techniques were reported as follows: 50%, 50%, 87.5%, 93.8%, and 93.8% in phase 1, phase 2, phase 3, phase 4, and phase 5. Yet, the recall values which obtained by the proposed model are like 53.1%, 50%, 90.6%, 96.9%, and 96.9% at the same phases in the same order. Notably, the proposed model provides better performance than the other ML techniques in the first, third, fourth, and fifth phases, and an equal recall values at the second phase . Similarly, in terms of AUC measure, the proposed model shows better performance than traditional ML techniques in the first, third, fourth, and fifth phases as shown in Fig. 3. However, in the second phase, SLR model gave better AUC results rather than the proposed model.

Further, to validate the proposed model in software product experiments, we compared the performance of the proposed model with the approach of Naseer, Zhang & Zhu (2020a). The related approach (Naseer, Zhang & Zhu, 2020a) demonstrated the same training and testing set like the proposed model, it applied J48 decision tree as classification model and information gain as a feature selection technique at the first five phases of software product development. The best recall values for their approach were 3.1%, 15.6%, 53.1%, 78.1%, and 75% at phase 1, phase 2, phase 3, phase 4, and phase 5 correspondingly. On the other hand, the best recall values for the proposed model are 53.1%, 50%, 90.6%, 96.9%, and 96.9% at phase 1, phase 2, phase 3, phase 4, and phase 5 respectively. Remarkably, the results reflect significant improvement of prediction that achieved by the proposed model.

In respect of the AUC values that obtained by the technique (Naseer, Zhang & Zhu, 2020a), the reported values of the AUC were 47.4%, 44%, 70.7%, 87%, and 85.4% in phase 1, phase 2, phase 3, phase 4, and phase 5. On the other side, the AUC values for the proposed model are 64.7%, 61.6% , 91.7%, 95.2%, and 96% in the same ordered phases.

Further, the F-Measure values for their approach were 6.1%, 26.3%, 64.2%, 87.7%, and 85.7% in phase 1, phase 2, phase 3, phase 4, and phase 5 whereas the F-Measure values of the proposed model are 57.6%, 51.6%, 90.6%, 93.9%, and 91.2% in the same phases. Significantly, the proposed model provides more reliable outcomes than the study (Naseer, Zhang & Zhu, 2020a) in terms of AUC and F-Measure.

Moreover, to compare TP rate and FP rate with the approach of Naseer, Zhang & Zhu (2020a), the values of (TP rate and FP rate) which reported in their approach were as (3.1% and 0%) at first phase, (15.6% and 2.4%) at second phase, (53.1% and 9.5%) at third phase, (78.1% and 0%) at fourth phase, and (75% and 0%) at fifth phase. On the other hand, the (TP rate and FP rate) values of the proposed model are reported as: (53.1% and 42.9%) at first phase, (50% 33.3%) at second phase, (90.6% and 7.1%) at third phase, (96.9% and 7.1%) at fourth phase, and (96.9% and 11.9%) at fifth phase respectively. Obviously, the proposed model shows an optimistic and superior predictability performance rather than the approach (Naseer, Zhang & Zhu, 2020a) at the investigated phases of software product development.

### Features analysis for the software product

The software product features captured the final software deliverable metrics and were collected from weekly time surveys, software tools logs, and instructor observation during the software project. It is important to detect the essential software product features that improve the overall success of software project. The SETAP dataset contains 115 product features. Based on obtained results, the number of selected product features are 18, 26, 33, 30, and 36 in the first, second, third, fourth, and fifth phases respectively. Table 7 shows the list of selected features at the first phase of software product development.

**Table 7:** The most relevant features at the first phase of software product development.

Number of selected features	18
	teamMemberCount
	teamDistribution
	helpHoursTotal
	leadAdminHoursAverage
	standardDeviationInPersonMeetingHoursTotalByWeek
	averageInPersonMeetingHoursAverageByWeek
	standardDeviationInPersonMeetingHoursAverageByWeek
Selected features	standardDeviationHelpHoursAverageByWeek
	averageGlobalLeadAdminHoursResponseCountByWeek
	standardDeviationMeetingHoursTotalByStudent
	averageHelpHoursAverageByStudent
	uniqueCommitMessageCount
	averageCommitCountByWeek
	standardDeviationUniqueCommitMessageCountByWeek
	averageUniqueCommitMessagePercentByWeek
	standardDeviationUniqueCommitMessagePercentByWeek
	averageCommitCountByStudent
	standardDeviationCommitMessageLengthAverageByStudent

The proposed model presents a better performance with a reduced number of software product features. For example, some of the key product features at the third phase are identified as: *averageUniqueCommitMessageCountByWeek*, *uniqueCommitMessageCount*, *commitMessageLengthStandardDeviation*, and *standardDeviationCodingDeliverablesHoursAverageByWeek*. The features *averageUniqueCommitMessageCountByWeek*, *uniqueCommitMessageCount*, and *commitMessageLengthStandardDeviation* are used to record statistics about average, total number, and length of the unique commit comment messages that related to code submission in software repository. In other words, high-expectation teams do proper commit messages which reflected by high average length, while low-expectation teams do not use new commit messages in each submission to the code repository instead they used non-unique messages. In addition, the *standardDeviationCodingDeliverablesHoursAverageByWeek* is used to measure the time taken for coding deliverable.

Comparing to the approach by Naseer, Zhang & Zhu (2020b), the mutual key product features across all the five investigated phases are: *FemaleTeamMembersPercent*, *averageInPersonMeetingHoursTotalbyWeek*, *averageMeetingHoursTotalByWeek*, *uniqueCommitMessageCount*, *averageGlobalLeadAdminHoursResponseCountByWeek*, *globalLeadAdminHoursResponseCount*, and *teamDistribution*.

The features like *averageInPersonMeetingHoursTotalbyWeek* and *averageMeetingHoursTotalByWeek* represent average time spent on meetings of each single team and for each single student which help to predict the coding progress for each team. Also, The features like *averageGlobalLeadAdminHoursResponseCountByWeek*, *globalLeadAdminHoursResponseCount*, and *teamDistribution* measure the progress for each global team and allow the instructors to properly identify the differences in cultural aspects, time zone, and physical distance as declared in Bello (2018) which might affect their efforts and their responsiveness to instructors.

Such product features used to collect statistical information of students teams to measure their performance. Such information capture different measurements like weekly meeting time for each software team and for each single student, code commits, number and length of commit messages by each week, local/global teams distribution, female proportion within a team, and total number of responses per each global team. Particularly, these features are important as they detect the key characteristics of software functionality, design, code deliverable, and effectiveness of final project delivery. Detecting such important product features would enable the software engineering instructors to observe low-expectation teams who deliver inconsistent software requirements, degraded design, poor code quality, and late commit messages.

### Software process results

The software process focuses on activities and practices perspectives of software team during software development life cycle. Regarding the software process development perspective. The previous studies used single phases of software process development in their experiments rather than considering the continues phases which help to monitor practices of low-expectation teams during the essential phases of SDLC.

This study aims to improve the performance prediction of low-expectation teams in the investigated phases of software process development to capture their dynamic behavior. Tables 8–12 exhibits the best performance achieved by PSO-1NN, PSO-5NN, and PSO-7NN with other traditional ML models in the investigated phases.

**Table 8:** Performance of the classifiers in the first phase for software process.

Algorithm	TP rate	FP rate	Precision	Recall	F-Measure	AUC	Accuracy
SMO	0	0	na	0	na	0.5	66.21
SLR	0	0	na	0	na	0.578	66.21
NB	0	0.041	0	0	na	0.499	63.51
MLP	0	0	na	0	na	0.579	66.21
Standard KNN	**0.918**	** 0.920**	0.662	**0.918**	0.769	0.499	63.51
PSO-1NN	**0.918**	0.76	**0.703**	**0.918**	**0.796**	**0.579**	**68.91**
PSO-5NN	0	0.02	0	0	0	0.396	64.86
PSO-7NN	0	0.02	0	0	0	0.45	64.86

**Notes.**

Numbers in bold indicate the best values.

By investigating the first phase of software development process, the proposed model outperform the other traditional ML classifiers in terms of accuracy, AUC, F-measure, and precision as presented in Table 8. In addition, standard KNN and the proposed model presented an equal recall value as (91.8%). Examining the second phase, the standard KNN mostly perform better than the proposed model as presented in Table 9. In the third phase, the proposed model performs better than ML models in the metrics of precision, recall, F-Measure, and AUC as shown in Table 10. As we proceed further, the proposed model began to produce better predictive outcomes. Interestingly, in the fourth phase, the proposed model provides better performance for all the used evaluation metrics than the other ML algorithms as shown in Table 11. Significantly, the trend also continues for the fifth phase as shown in Table 12. Undoubtedly, this interesting findings reveals that the proposed model provides better results as moving to further phases of software process development. Such improvement is reasonable as the later phases convey a better understanding of teamwork behaviors in comparing with the prior phases.

**Table 9:** Performance of the classifiers in the second phase for software process.

Algorithm	TP rate	FP rate	Precision	Recall	F-Measure	AUC	Accuracy
SMO	0.56	0.204	0.583	0.56	0.571	0.678	71.62
SLR	0.6	0.143	0.682	0.6	0.638	0.745	**77.027**
NB	0.2	0.082	0.556	0.2	0.294	0.712	67.57
MLP	0.28	0.082	0.636	0.28	0.389	0.769	70.27
Standard KNN	**0.878**	**0.600**	0.741	**0.878**	**0.804**	0.639	71.62
PSO-1NN	0.44	0.163	0.579	0.44	0.5	0.633	70.27
PSO-5NN	0.36	0.061	**0.75**	0.36	0.486	0.761	74.32
PSO-7NN	0.36	0.061	**0.75**	0.36	0.486	**0.784**	74.32

**Notes.**

Numbers in bold indicate the best values.

**Table 10:** Performance of the classifiers in the third phase for software process.

Algorithm	TP rate	FP rate	Precision	Recall	F-Measure	AUC	Accuracy
SMO	0.52	0.082	0.765	0.52	0.619	0.719	78.38
SLR	0.44	0.061	0.786	0.44	0.564	0.764	77.027
NB	0.4	**0.327**	0.385	0.4	0.392	0.642	58.11
MLP	0.84	0.102	0.808	0.84	0.824	0.819	**87.84**
Standard KNN	0.755	0.240	0.860	0.755	0.804	0.758	75.67
PSO-1NN	**0.939**	0.28	**0.868**	**0.939**	**0.902**	**0.829**	86.48
PSO-5NN	0.28	0.082	0.636	0.28	0.389	0.761	70.27
PSO-7NN	0.16	0.041	0.667	0.16	0.258	0.739	68.92

**Notes.**

Numbers in bold indicate the best values.

**Table 11:** Performance of the classifiers in the fourth phase for software process.

Algorithm	TP rate	FP rate	Precision	Recall	F-Measure	AUC	Accuracy
SMO	0.44	0.061	0.786	0.44	0.564	0.689	77.027
SLR	0.52	0.041	0.867	0.52	0.65	0.863	81.08
NB	0.24	0.122	0.5	0.24	0.324	0.53	66.22
MLP	0.56	0.061	0.824	0.56	0.667	0.794	81.08
Standard KNN	0.857	0.240	0.875	0.857	0.866	0.809	82.43
PSO-1NN	**0.939**	0.12	**0.939**	**0.939**	**0.939**	**0.909**	**91.89**
PSO-5NN	0.6	0.061	0.833	0.6	0.698	0.904	82.43
PSO-7NN	0.52	**0.61**	0.813	0.52	0.634	0.0.863	79.7297

**Notes.**

Numbers in bold indicate the best values.

**Table 12:** Performance of the classifiers in the fifth phase for software process.

Algorithm	TP rate	FP rate	Precision	Recall	F-Measure	AUC	Accuracy
SMO	0.52	0.061	0.813	0.52	0.634	0.729	79.73
SLR	0.56	0.061	0.824	0.56	0.667	0.898	81.08
NB	0.44	0.163	0.579	0.44	0.5	0.754	70.27
MLP	0.8	0.204	0.667	0.8	0.727	0.837	79.73
Standard KNN	0.898	0.120	0.936	0.898	0.917	0.889	89.18
PSO-1NN	**0.939**	0.12	**0.939**	**0.939**	**0.939**	0.909	**91.89**
PSO-5NN	0.72	0.061	0.857	0.72	0.783	**0.919**	86.487
PSO-7NN	0.56	**0.61**	0.824	0.56	0.667	0.901	81.08

**Notes.**

Numbers in bold indicate the best values.

In terms of recall metric for the investigated phases of software process development. The results reflect an equal performance in the first phase for both as achieved by standard KNN and PSO-1NN. However, at the second phase, standard KNN classifier outperforms better than the proposed model, this would be referred to the reason that low-expectation teams hardly establish communication and coordination while they working on design phase. Results indicated that later phases reflect much stronger communication skills comparing to the prior phases as a better performance was achieved in the implementation, testing, and maintenance phases. The best values for ML techniques and the proposed model were reported as (91.8%, 91.8%) in phase 1, (75.5%, 93.9%) in phase 3, (85.7%, 93.9%) in phase 4, and (89.8%, 93.9%) in phase 5. Obviously, the proposed model exhibited better performance rather than the ML techniques at the third, fourth, and fifth phases, and an equal performance at the first phase.

In particular to precision metric, the best precision values obtained by traditional ML were 66.2%, 74.1%, 86%, 87.5%, and 93.6% at phase 1, phase 2, phase 3, phase 4, and phase 5. The best precision values achieved by the proposed model were reported as 70.3%, 75%, 86.8%, 93.9%, and 93.9% at the same phases in same order. Clearly, the proposed model shows better precision values than the ML techniques across all the investigated phases.

**Figure 4:**
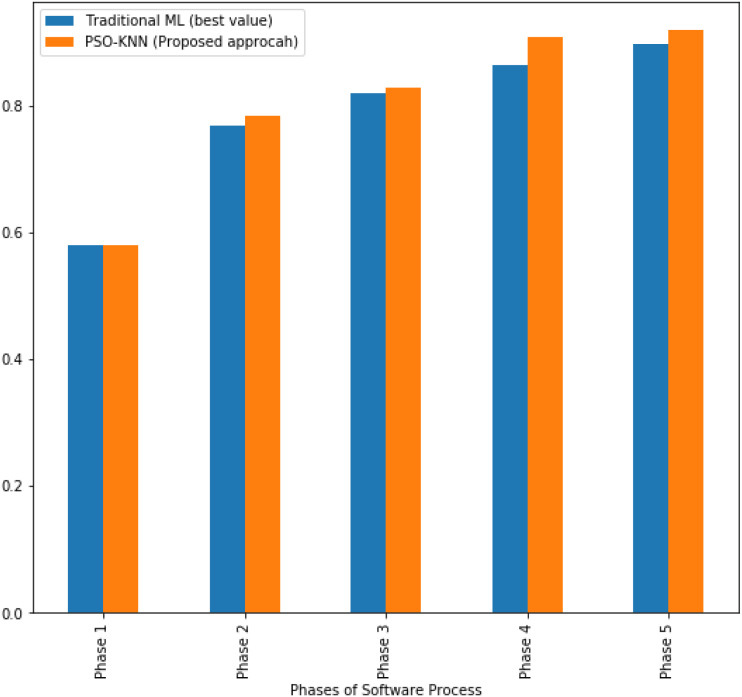
The AUC values obtained by the proposed model and the best values achieved by ML techniques for software process.

In addition, with respect to AUC measure, the proposed model shows better performance rather than traditional ML techniques in the all investigated phases as shown in Fig. 4. Also, regarding to the accuracy metric, the best achieved accuracy values for ML models and the proposed models were outlined as (66.2% and 68.9%) at phase 1, (82.4% and 91.8%) at phase 4, and (89.1% and 91.8%) at phase 5. The proposed model revealed better performance than other ML classifiers at the first, fourth, and fifth phases of software process development.

Generally, comparing with PSO-5NN and PSO-7NN, PSO-1NN achieved the best performance results in the most investigated phases of software product and process development. The accuracy average and standard deviation values of PSO-1NN model of software product and process can be found in Tables 13 and 14.

**Table 13:** Accuracy average and standard deviation results of all investigated phases for software product.

Datasets	PSO-1NN	
	**Avg**	**Std**
First phase (Product)	57.66	7.69
Second phase (Product)	60.81	4.05
Third phase (Product)	88.74	5.46
Fourth phase (Product)	93.69	1.56
Fifth phase (Product)	89.64	2.82

**Table 14:** Accuracy average and standard deviation results of all investigated phases for software process.

Datasets	PSO-1NN	
	**Avg**	**Std**
First phase (Process)	65.76	5.46
Second phase (Process)	70.72	0.78
Third phase (Process)	81.08	7.15
Fourth phase (Process)	90.54	1.36
Fifth phase (Process)	88.73	2.82

### Features analysis for the software process

The software process features measure activities and practices of software team during software project. The process features used to collect information about behavior characteristics of software team during software development process. The SETAP dataset contains 84 process features. Based on our results, the number of selected process features are 16, 26, 34, 28, and 25 in phase 1, phase 2, phase 3, phase 4, and phase 5 correspondingly. Table 15 shows the list of selected features at the first phase of software process development.

**Table 15:** The most relevant features at the first phase of software process development.

Number of selected features	16
	teamMemberCount
	femaleTeamMembersPercent
	teamMemberResponseCount
	meetingHoursAverage
	meetingHoursStandardDeviation
	nonCodingDeliverablesHoursAverage
**Selected features**	averageMeetingHoursTotalByWeek
	averageMeetingHoursAverageByWeek
	standardDeviationMeetingHoursAverageByWeek
	averageInPersonMeetingHoursTotalByWeek
	averageResponsesByStudent
	standardDeviationMeetingHoursAverageByStudent
	averageInPersonMeetingHoursAverageByStudent
	commitCount
	commitMessageLengthTotal
	averageCommitMessageLengthTotalByWeek

The proposed model attempts to select the most relevant process features. For example, some of the key process features at the second phase are stated like: *issueCount*, *lateIssueCount*, *helpHoursStandardDeviation*, *averageNonCodingDeliverablesHoursAverageByWeek*, and *averageResponsesByWeek*. Such features help to check behavior aspects of team activities during a certain period of time. Time is one of the most critical process features to evaluate the performance in the software project. The features *meetingHoursTotal* and *inPersonMeetingHoursAverage* represents total and average for time spent on meetings for each team to detect their participation/practices through meetings. The *helpHoursTotal* is calculated by counting required time that spent for obtaining help for unclear issues or other challenges, the *averageNonCodingDeliverablesHoursAverageByWeek* measures average time taken for delivering non-coding parts as documentation. In addition, other features like *onTimeIssueCount* and *lateIssueCount* used to measure the completeness/lateness of submission to help the instructor observe software teams who have on time and late delivery. Further, the *averageResponsesByWeek* is used to evaluate the ability of teams to response for instructor requests and deal with feedback constructively. Detecting such key process features help to evaluate behavior of students teams by measuring different activities aspects such as team participation, using of communication tools, and the time needed to submit the software deliverables.

## Discussion

Software engineering is an important field that aims to apply software processes and methodologies in education and industry environment to deliver software product. Software Engineering education involves teaching software projects for students in universities (Ahtee & Poranen, 2007). Students must work in teams in projects and should obtain above-average grades in order to successfully pass the course. Software product and process features are measures that outline the level of performance of each team in different phases of SDLC. Each team is evaluated at theoretical and technical perspective based on multiple phases of assessments to detect performance of students during different stages of software project. The theoretical aspect reflects the software process aspects while the technical aspect is relevant to the software product aspects. Teams should complete all the required parts of the software deliverables but might have different grades between product and process parts based on their knowledge levels and their practical skills, certain types of teams may perform better in one part compared to the other one. Software teams are either distributed globally or locally based on the project nature in software engineering course. Software teams faced many challenges that caused to deliver improper project like temporal, cultural, geographical distances, cooperation difficulties, communication issues, assigning roles within team members, incorrect software deliverables, delay submission , and adapting wrong practices. These challenges have significant effects on the project progress and the final grade for each team. As a result, it is necessary to teach the students about the proper methodologies of software development to get better grades.

Clearly, it is hard to predict the performance of software teams in software projects due to the aforementioned challenges and difficulties (Chan et al., 2017). Predicting the performance of students teams is one of the most important tasks for learning of software projects in software engineering education. The early prediction of low-expectation teams involves the implementing of ML classification models and focused essentially on estimating the behaviors of teams at early stages of software project. In practice, the influence of instructors and their teaching strategy could be resulted in a significant performance variation among the software teams. Therefore, a special attention should be paid to the performance of low-performing teams. This paper proposes an swarm intelligence -based model which mainly aims to improve the prediction performance of low-expectation teams at the earliest possible phases of SDLC by capturing both of product and process perspectives of software project.

By using the proposed model, the software engineering instructors could detect misunderstandings and challenges related to low-expectation teams at the earliest possible phases of software project which helps to decrease the ratio for software project failure. In terms of software process development, software engineering instructors can track the practical behavior and activities of teams during they work on software product deliverables which enable to early detect any difficulties of project tasks in order to improve cooperative learning among teams members. The swarm intelligence-based model would help instructors better assess software teams’ performance by capturing the correctness and time-completeness for the project parts. The correctness reflects the quality of the submitted deliverables whereas the time-completeness is used to distinguish between the on-time and late submission for these deliverables.

Software industry requires improvements in learning skills in the software engineering education field (Ramirez-Mendoza et al., 2020; Cico et al., 2021). As the proposed model captures both software and product perspectives to improve the prediction performance for low-expectation teams at the earliest possible phases of SDLC to early avoid project failure. Improving the learning practices and outcomes of software projects would encourage the low-expectation teams to progress in more efficient manner and prepare them to better move for software industry at their future careers. This model provides a good opportunity to explore the mutual area between software industry and software engineering education by increasing the possibilities of success for low-expectation teams in software projects. The proposed model would be considered to have a significant implication at theoretical (*i.e.,* software product) and practical aspects (*i.e.,* software process) of software project.

Experiments have been demonstrated on the SETAP dataset to validate the proposed model, software product and process features in this dataset allow instructors to detect the performance of software teams through different stages of assessments during the academic semester. In terms of software product experiments, the proposed assessment model outperforms the traditional ML techniques and the related studies at the first, third, fourth, and fifth phases, and it shows a slightly acceptable performance at the second phase. Similarly, with respect to software process experiments, the proposed model shows better prediction at the first phase and provides an acceptable prediction in the second phase. Interestingly, the proposed model reveals much better predictions at the third, fourth and fifth phases comparing with the used traditional ML techniques.

Feature selection is an urgent task when there are a large number of features in the training data as in the investigated dataset of SETAP project. Feature selection helps to allow quick learning for ML classifier and increase the accuracy for the prediction model by reducing the selected features as well as it minimizes the cost of ML classifiers. Consequently, the proposed assessment model attempts to reduce the total number of features to reach higher accuracy with identifying less than 40 features of software product and process. As the value of (K) parameter has a significant impact on classification performance. Three different values of (K) were selected based on studying the most related literature and the previous works to construct three combinations as PSO-1NN, PSO-5NN, and PSO-7NN. Mostly, the PSO-1NN gave superior results for both software product and process development as reported in our findings.

The software product features considers characteristics of each software deliverable at each phase of SDLC. The software process features are relevant to the dynamic behavior of teams and they reflect the collaboration and communication rate within the team members. In the light of software product and process features, the selected software product and process features are used for model training while the target variable (*i.e.,* grade) is used for evaluating performance for low-expectation teams at each investigated phase of software project. Obviously, using the (PSO) as a feature selection technique in the proposed model leads to improve the prediction performance in the investigated phases. The findings which obtained by implementing PSO show a significant improvement comparing with the findings that applied only traditional ML classifiers.

## Limitations

We acknowledge that this study has some limitations. First, It is difficult to generalize the reported findings of the proposed model on one type of datasets, different types of dataset were difficult to obtain in software engineering education. Second, the proposed model demonstrated the experiments of software product and process parts separately, in case of combining the product and process features together, the prediction performance of the proposed model might be affected.

## Conclusion and Future Work

Software engineering is one of the most significant areas which extensively used for both education and industry fields. Software project is considered as a core part of software engineering course. Software project includes composition of software product and process parts. The low-expectation teams face challenges during different stages of software project. The early prediction of performance for these teams help instructors to identify the difficulties and challenges at early phases of software project to avoid project failure. This paper proposed an swarm intelligence -based model which aims to improve the prediction performance for low-expectation teams at the earliest possible stages of software product and process development. Experiments were demonstrated on the public SETAP project dataset. The proposed model was compared with the state-of-the-art machine learning (ML) classifiers: Sequential Minimal Optimization (SMO), Simple Linear Regression (SLR), Naïve Bayes (NB), Multilayer Perceptron (MLP), standard KNN, and J48. Mostly, the proposed model provides superior results compared to the traditional ML classifiers and state-of-the-art studies. Besides, the proposed model attempts to reduce the total number of features to reach higher accuracy with identifying less than 40 features instead of considering all features of software product and process.

As a future work, the proposed model would open the doors for further researching in the software process development. Another future direction is the possibility to apply the proposed model on other types of dataset in software engineering education and software industry. Future research would be valuable to study the ability to apply the proposed model on combination of software product and process features. Besides using the PSO-KNN model, there is a room for exploring the prediction accuracy of performance for low-expectation teams by combining PSO with other classifiers such as NB, MLP, and etc. Such efforts are best left to be done in the future. In addition, this study could be extend to build software tool that would help instructors to automatically predict the performance of software teams in both of software product and process development. Such an extension would be considerable for ease of comparison with the existing software tools in the literature.

## Additional Information and Declarations

### Data Availability

The following information was supplied regarding data availability: The data is available at the UCI Machine Learning Repository [11-6-2020]: https://archive.ics.uci.edu/ml/datasets/Data+for+Software+Engineering+Teamwork+Assessment+in+Education+Setting
The code is available at GitHub: https://github.com/aljarrahcs/EvoloPy-FS.
